# Antioxidant and prebiotic activity of five peonidin-based anthocyanins extracted from purple sweet potato (*Ipomoea batatas* (L.) *Lam*.)

**DOI:** 10.1038/s41598-018-23397-0

**Published:** 2018-03-22

**Authors:** Hanju Sun, Pingping Zhang, Yongsheng Zhu, Qiuyan Lou, Shudong He

**Affiliations:** 1 School of Food Science and Engineering, Hefei University of Technology, Hefei, 230009 Anhui PR China; 2 School of Food Science and Technology, National Engineering Research Center of Seafood, Dalian Polytechnic University, Dalian, 116034 PR China

## Abstract

Twelve kinds of anthocyanins from the Chinese purple sweet potato cultivar (*Ipomoea batatas* (L.) *Lam*.) were extracted and identified using LC-MS/MS, which had a high content of peonidin-based anthocyanins. Five peonidin-based anthocyanin monomers (P1, P2, P3, P4 and P5) were isolated by preparative liquid chromatography with structural analyses using an Impact II Q-TOF MS/MS. Then, the functional properties of the anthocyanin monomers, such as the antioxidant activities, proliferative effects on probiotics, and their inhibition on harmful bacteria *in vitro*, were investigated. The peonidin-based components in purple sweet potato anthocyanins (PSPAs) showed good properties regarding scavenging 1,1-diphenyl-2-picrylhydrazyl (DPPH) radicals and superoxide anions, and had good potential in reducing the total power activity and Fe^2+^ chelating ability. While the order of the antioxidant abilities was as follows: P4 > P5 > P3 > P2 > P1 > PSPAs. Microbial cultivations showed that P1, P2, P3, P4, P5 and PSPAs could induce the proliferation of *Bifidobacterium bifidum*, *Bifidobacterium adolescentis*, *Bifidobacterium infantis* and *Lactobacillus acidophilus*, and they inhibited the growth of *Staphylococcus aureus* and *Salmonella typhimurium*, suggesting the anthocyanins might have prebiotic-like activity through the modulation of the intestinal microbiota. Our results indicate that peonidin-based anthocyanins could be further utilized in health foods and pharmaceutical developments.

## Introduction

Anthocyanins are natural pigments belonging to the flavonoid family and are responsible for a wide range of colors in fruits, vegetables and flowers. They are polyhydroxy or polymethoxy derivatives of 2-phenyl-benzopyrylium and most of them are present in plants attached to sugars as mono, di or triglycosides by α- or β-linkages^1^. There are more than 500 anthocyanins in nature, and the most common anthocyanins in plants are cyanidin (Cn), pelargonidin (Pg), peonidin (Pn), delphinidin (Dp), petunidin (Pt) and malvidin (Mv), which have a 3-glycoside structure^2^. Due to the urgent need for natural and healthy pigments in the food industry, the exploration of anthocyanins from edible plants is receiving increasing attention^3^.

Purple sweet potato (PSP), also known as Okinawan potato, is a new kind of sweet potato that is low in carbohydrates and fats and rich in fiber and vitamins, and it is widely cultivated in South America and Asian countries, especially in China, Korea and Japan. The PSP has many varieties, including *Ipomoea batatas* (L.) *Lam*., which is cultured in the subtropical regions of China, such as Fujian, Guangxi, and Jiangxi. It is noteworthy that PSP contains high anthocyanin content, and the purple sweet potato anthocyanins (PSPAs) are more color stable than those extracted from strawberry, red cabbage, perilla and other plants, due to their special mono- or di-acylated forms^4^. Recently, a total of fifteen individual PSPAs from a Korean variety, named Shinzami, were identified using the LC-DAD-ESI/MS method, and the primary component was peonidin-based anthocyanin^5^. Twelve kinds of PSPAs from ten Chinese genotypes cultivated in Hubei, Jiangsu and Chongqing, were characterized by liquid chromatography–photodiode array detector–atmospheric pressure chemical ionization−mass spectrometry (LC–PDA–APCI–MS), while the peonidin or cyanidin 3-sophorsoside-5-glucoside and their acylated derivatives were confirmed as major anthocyanins^6^. However, there has been little systematic information on the structural and chemical compositions of PSPAs from *Ipomoea batatas* (L.) *Lam*.

PSP might have diverse biological activities, including anticancer, antidiabetic, anti-inflammatory, anti-bacteria, and hepatoprotective activities^7^, but these activities have not been proven or validated, and many of these activities might be attributed to the potent antioxidant activity of anthocyanins with the two accepted mechanisms of hydrogen atom transfer and single-electron transfer followed by proton transfer^8,9^. Furthermore, the structural features of anthocyanins, such as the disparity in the kind, number, and position of hydroxyl and methoxy substituents as well as the electron donating groups on the flavylium ion, should be of more concern^10^.

Meanwhile, recent studies have shown that polyphenols and their metabolites have potentially beneficial effects on the intestinal bacterial flora, especially on the growth of *Lactobacillus spp*. and *Bifidobacteria spp*., which are the predominant probiotics in the intestinal tract. Seven bacteria (*Bacillus megaterium DSM 32*, *Pseudomonas aeruginosa DSM 9027*, *Staphylococcus aureus Cowan 1*, *Corynebacterium xerosis UC 9165*, *Escherichia coli DM*, *Enterococcus faecalis A10*, *Micrococcus luteus LA 2971*), and three fungi (*Kluyveromyces marxianus A 230*, *Rhodotorula rubra MC 12*, *Candida albicans ATCC 1023*) were reported to be inhibited by the polyphenol extracts from pomegranates (*Punica granatum L*)^11^. Meanwhile, crude PSPAs were found to impede the growth of *Bacteroides*, *Prevotella* and *Clostridium histolyticum* in previous studies^12^.

As far as our literature survey ascertained, information on the antioxidant and probiotic activities of the purified anthocyanin monomers were rarely available hitherto. Thus, the overall objectives of this study were: (1) to identify the structure of anthocyanins from the Chinese PSP (*Ipomoea batatas* (L.) *Lam*.) cultivar, (2) and to purify the main anthocyanin monomers of peonidin derivatives, (3) as well as to investigate the antioxidant and probiotic activities of specific peonidin-based anthocyanin(s). Our research could provide a better understanding of anthocyanins from PSP and promote its further investigation and application in the food industry and in medical treatments.

## Results

### HPLC analysis and LC-MS/MS identification

**Figure 1:**
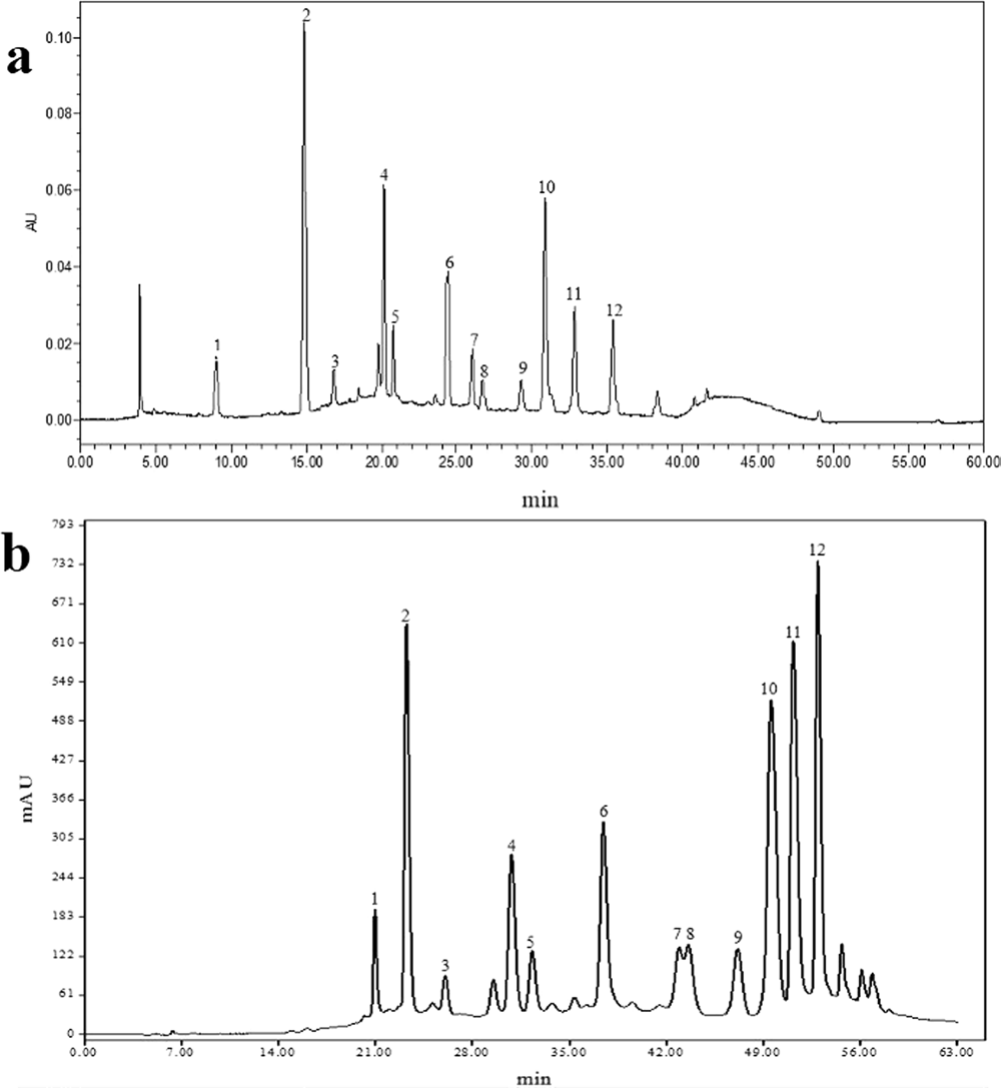
HPLC profile of the anthocyanins extracted from PSP (**a**), and preparative chromatographic separation of anthocyanins in PSP by gradient elute at 520 nm (**b**).

The high-performance liquid chromatographic (HPLC) fingerprints of anthocyanins extracted from PSP are shown in Fig. 1(a). Twelve kinds of anthocyanin peaks were obviously observed, and six main peaks 2, 4, 6, 10, 11 and 12, which were eluted after 14.874, 20.143, 24.368, 30.841, 32.809 and 35.356 min, accounted for 25.92, 9.41, 8.36, 18.01, 8.60 and 7.76% of the total anthocyanins (Table 1), respectively, by HPLC peak integration.

**Table 1:** Chromatographic characteristics and speculative structures of 12 kinds of anthocyanins in PSP by LC-MS/MS analysis.

Peak no.	Retention time (min)	Relative content (%)	M^+^ (m/z)	MS/MS (m/z)	Speculative structures
1	9.016	4.41	773	611, 449, 287	cyanidin-3-sophoroside-5-glucoside
2	14.874	25.92	787	625, 463, 301	peonidin-3-sophoroside-5-glucoside
3	16.815	2.94	893	731, 449, 287	cyanidin-3-p-hydroxy benzoylsophoroside-5-glucoside
4	20.143	9.41	907	745, 463, 301	peonidin-3-p-hydroxy benzoylsophoroside-5-glucoside
5	20.759	3.07	949	787, 449, 287	cyanidin-3-feruloylsophoroside-5-glucoside
6	24.368	8.36	963	801, 463, 301	peonidin-3-feruloyl sophoroside-5-glucoside
7	26.034	3.61	935	773, 449, 287	cyanidin-3-caffeoyl sophoroside-5-glucoside
8	26.711	1.92	1055	893, 449, 287	cyanidin-3-caffeoyl-p-hydroxy benzoyl sophoroside-5-glucoside
9	29.273	2.06	1111	949, 449, 287	cyanidin-3-caffeoyl-feruloyl sophoroside-5-glucoside
10	30.841	18.01	949	787, 463, 301	peonidin-3-caffeoyl sophoroside-5-glucoside
11	32.809	8.60	1069	907, 463, 301	peonidin-3-caffeoyl-p-hydroxy benzoyl sophoroside-5-glucoside
12	35.356	7.76	1125	963, 463, 301	peonidin-3-caffeoyl-feruloyl sophoroside-5-glucoside

Peak no. in this table refers to the numbers of peaks in Fig. 1(a).

Each peak was submitted to LC-MS/MS subsequently for the anthocyanin identification based on the retention times and mass spectral data in the library. As shown in Table 1, a range of positive molecular ion [M^+^] from m/z 773 to1125 was obtained in the PSPAs. According to the fragment patterns and previous reports^13^, twelve anthocyanins were identified as cyanidin-3-sophoroside-5-glucoside (peak 1), peonidin-3-sophoroside-5-glucoside (peak 2), cyanidin-3-p-hydroxy benzoyl sophoroside-5-glucoside (peak 3), peonidin-3-p-hydroxy benzoyl sophoroside-5-glucoside (peak 4), cyanidin-3-feruloyl sophoroside-5-glucoside (peak 5), peonidin-3-feruloyl sophoroside-5-glucoside (peak 6), cyanidin-3-caffeoyl sophoroside-5-glucoside (peak 7), cyanidin-3-caffeoyl-p-hydroxy benzoyl sophoroside-5-glucoside (peak 8), cyanidin-3-caffeoyl-feruloyl sophoroside-5-glucoside (peak 9), peonidin-3-caffeoyl sophoroside-5-glucoside (peak 10), peonidin-3-caffeoyl-p-hydroxy benzoyl sophoroside-5-glucoside (peak 11) and peonidin-3-caffeoyl-feruloyl sophoroside-5-glucoside (peak 12), respectively. And the main peaks (2, 4, 6, 10, 11 and 12) were speculated as the peonidin-types.

Then, the main peaks were further purified using the preparative HPLC (Fig. 1(b)), and the purities for peaks 2, 6, 10, 11 and 12 proved to be more than 98% by analytical HPLC, except for peak 4, which had impure peaks during the purification process. Thus, the anthocyanin monomer peaks 2, 6, 10, 11 and 12 were abbreviated as P1, P2, P3, P4 and P5, respectively, for further structural identifications by the tandem MS (MS/MS) spectral analysis.

**Figure 2:**
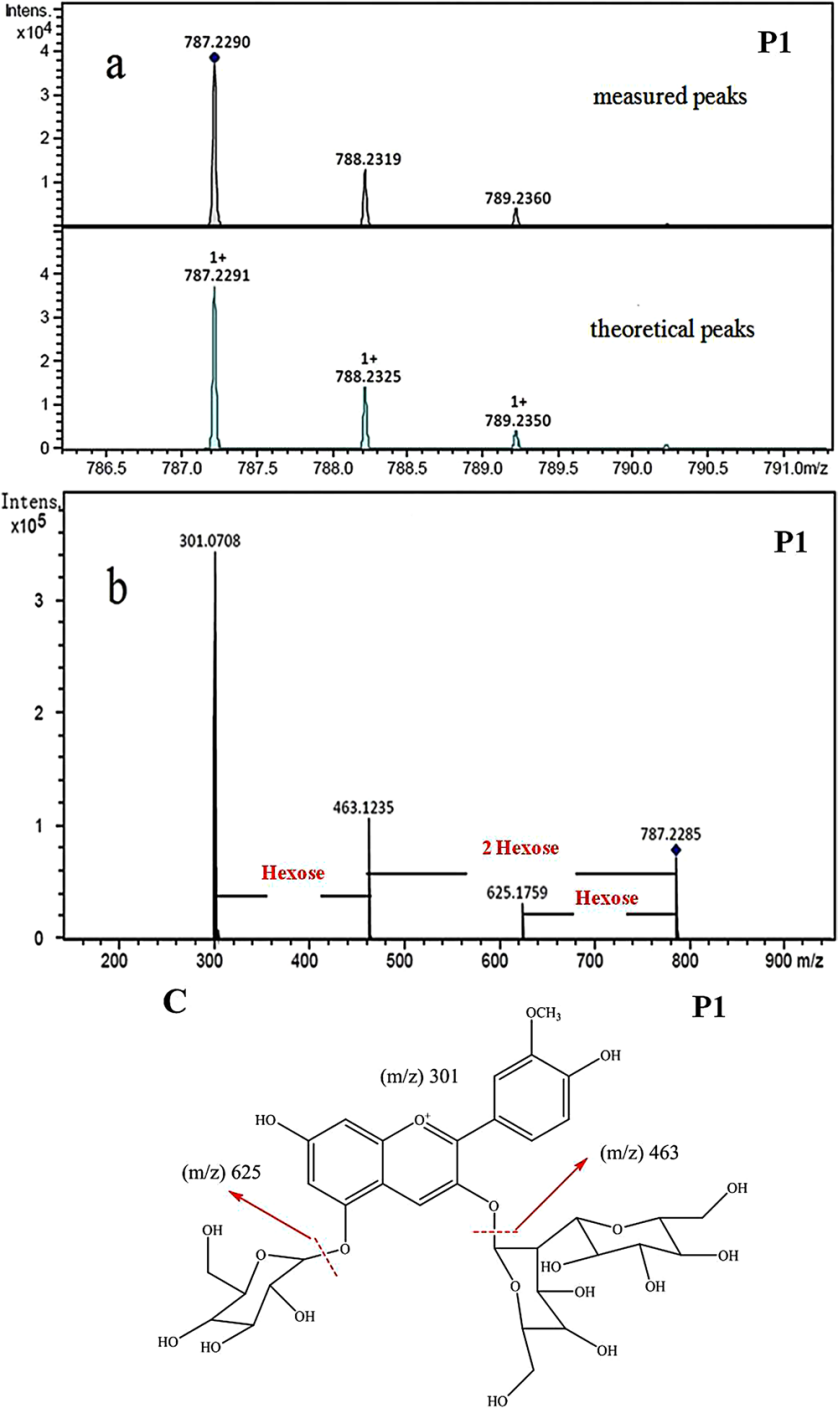
Mass spectrometric data and chemical structure of peonidin-based anthocyanins. (**a**) HPLC-TOF-MS spectrum of P1 (peak 2), (**b**) HPLC-TOF-MS/MS spectrum of P1 (peak 2), (**c**) chemical structure of P1 (peak 2).

The MS spectra, MS/MS spectra and chemical structures of the peonidin-based anthocyanins are shown in Figs 2 and S1–S4. The quasi-molecular ion ([M]^+^) data of *m/z* 787.2290 was obtained in the LC-TOF/MS analysis in peak 2 (Fig. 2(a)), which was very close to the theoretical value (*m/z* 787.2291) with an error of less than 1.5 ppm, confirming the veracity of the molecular structure analysis. As shown in Fig. 2(b), three ions were further fragmented from the [M]^+^ by LC-MS/MS method, and the fragment ion m/z 625 [M-162]^+^ indicated the loss of a glucoside residue, *m/z* 463 [M-324]^+^ indicated the loss of a sophoroside residue, as well as *m/z* 301 [M-(162+324)]^+^ was the diagnostic fragment of peonidin^5^. Thus, the peak 2 compound could be identified as peonidin-3-sophoroside-5-glucoside (Fig. 2(c)). An [M]^+^ ion at *m/z* 963 was observed in the MS spectrum of peak 6 (Supplementary Fig. S1), and the typical fragment ions at *m/z* 801 [M-162]^+^, 463 [M-(324+176)]^+^ and 301 [M-162-(324+176)]^+^ were obtained by fragmentation, which attributed to the loss of glucoside, sophoroside with feruloyl residues, and peonidin, respectively^14^. Thus, peak 6 was identified as peonidin-3-feruloyl sophoroside-5-glucoside. C_43_H_49_O_24_ might be suggested to be the basic molecular formula of peak 10 because of the [M]^+^ ion at *m/z* 949.2606 (Supplementary Fig. S2)^13^. The molecular ion of peak 10 was fragmented into three ions at *m/z* 787 [M-162]^+^, 463 [M-(324+162)]^+^ and 301 [M-162-(324+162)]^+^, which were calculated as glucoside, caffeoyl sophoroside and peonidin, respectively^14^. Then, peak 10 could be identified as peonidin-3-caffeoyl sophoroside-5-glucoside. The [M]^+^ ion at *m/z* 1069.2816 was given by peak 11 in the MS spectrum (Supplementary Fig. S3), and was identified as peonidin-3-coffeoyl-p-hydroxybenzoyl sophoroside-5-glucoside according to the fragment ions at *m/z* 907 [M-162]^+^, 463 [M-(324+162+120)]^+^ and 301 [M-162-(324+162+120)]^+^, which correspond to glucoside, caffeoyl-p-hydroxy benzoyl sophoroside and peonidin, respectively^13^. The measured mass value for peak 12 was *m/z* 1125.3100 (Supplementary Fig. S4), while the tandem fragment ions from peak 12 were at *m/z* 963 [M-162]^+^, 463 [M-(324+176+162)]^+^ and 301 [M-162-(324+176+162)]^+^, which correspond to glucoside, caffeoyl-feruloyl sophoroside and peonidin, respectively. Thus, peak 12 was ascertained as peonidin-3-caffeoyl-feruloyl sophoroside-5-glucoside.

### *In vitro* antioxidant activity

**Figure 3:**
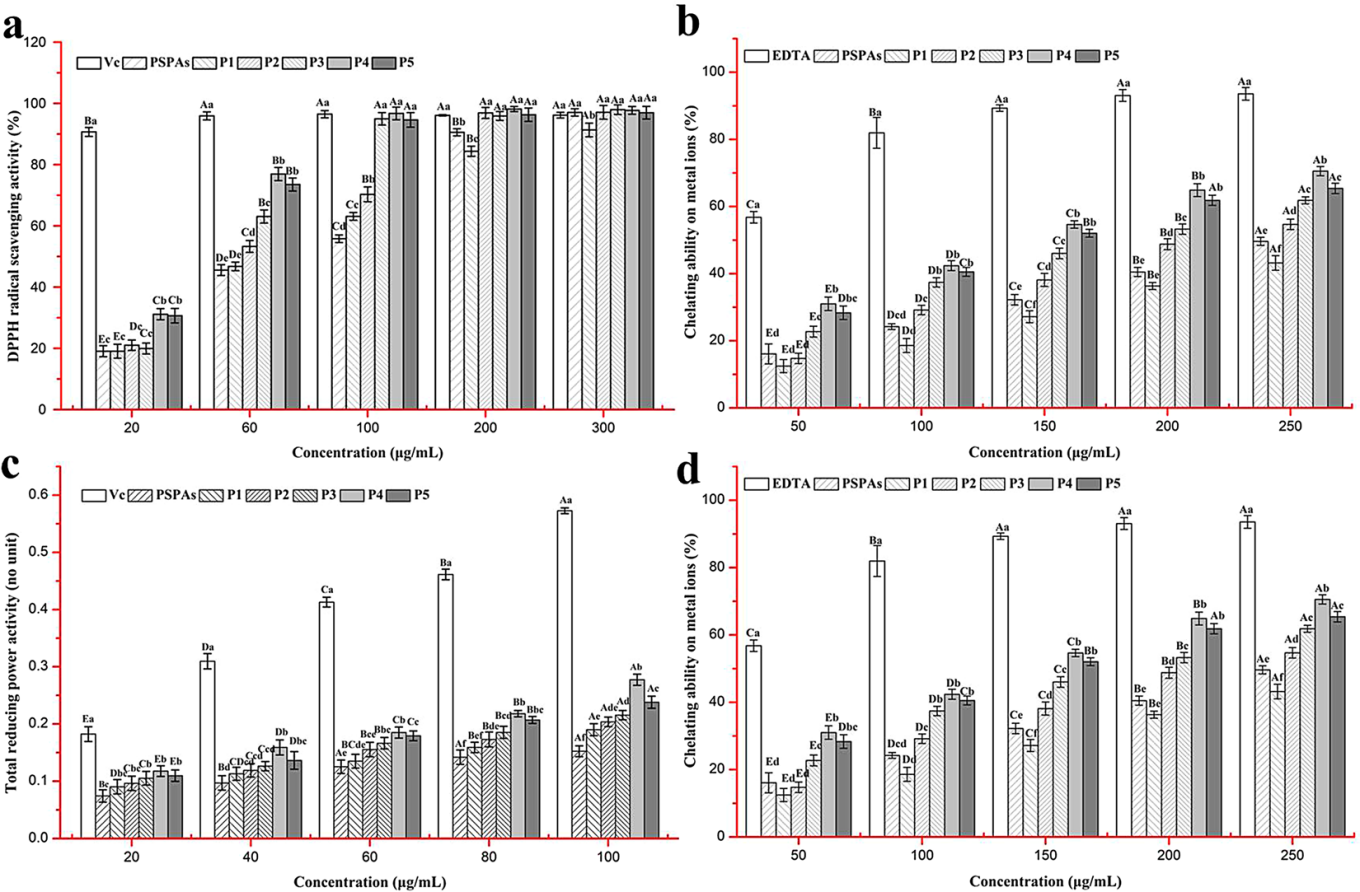
*In vitro* antioxidant activities of PSPAs and peonidin-based anthocyanin monomers (P1, P2, P3, P4 and P5). (**a**) DPPH radical-scavenging ability, (**b**) superoxide anion radical (O_2_^−^·) scavenging ability, (**c**) total reducing power, (**d**) chelating ability. Vc was used as positive control in DPPH radical-scavenging ability, superoxide anion radical (O_2_^−^·) scavenging ability and total reducing power evaluations. EDTA was used as positive control in the chelating ability evaluation. Different lowercase letters indicate significant differences (*P* < 0.05) among PSPAs and peonidin-based anthocyanin monomers at same concentration. Different capital letters indicate significant differences (*P* < 0.05) among different sample concentrations in each sample group.

The DPPH· scavenging capacities of PSPAs and PSP peonidin-based anthocyanin monomers are shown in Fig. 3(a). Compared with vitamin C (Vc) control, a significant increase of DPPH radical scavenging acidity was observably obtained with the increase in the concentration of anthocyanins (both the PSPAs and anthocyanin monomers), which suggested that the % DPPH· quenched value was depended on the concentration of the peonidin-based anthocyanins at a certain reaction time. The IC_50_ values of the PSPAs, P1, P2, P3, P4 and P5 were 57.58, 61.07, 47.22, 37.52, 26.71 and 28.76 μg/mL, respectively, indicating that peonidin-based anthocyanin monomers had a stronger DPPH· scavenging activity.

The active oxygen (O^2−^) scavenging activities of the PSPAs and PSP peonidin-based anthocyanin monomers have been presented in Fig. 3(b). Although the scavenging activities of anthocyanins were weaker than that of Vc at 20 μg/mL, excellent superoxide anion-scavenging activities were shown with increased concentrations of anthocyanins, and the IC_50_ values of the PSPAs, P1, P2, P3, P4 and P5 were obtained at 87.08, 57.88, 45.14, 37.08, 29.05 and 30.62 μg/mL, respectively.

The total reducing power of the peonidin-based anthocyanins was measured by the increase in the absorbance at 700 nm and is shown in Fig. 3(c). It was obviously found that the TRP of anthocyanins increased with an increase in the concentrations. Good TRP values from 0.11 to 0.28 were observed in the peonidin-based anthocyanin concentration range between 20 and 100 μg/mL, indicating that the antioxidant concentration might determine the electron donating ability, which could reduce the ferric ion complex to the ferrous form. Meanwhile, the highest absorbance was obtained in the P4 group, which had the same activity rank as the superoxide anion radical scavenging activity.

As presented in Fig. 3(d), the metal chelating activity increased depending on the increase in the concentration of both the PSPAs and anthocyanin monomers. Although the ferrous ion-chelating activities of anthocyanins were significantly lower than that of EDTA, the acylated anthocyanins expect P1 (IC_50_ value of 353.23 μg/mL) exhibited stronger chelating effects, as the IC_50_ values of P4, P5, P3, P2 reached 116.95, 133.06, 167.27 and 213.74 μg/mL, respectively, which were lower than the IC_50_ of PSPAs (287.85 μg/mL).

### Effects of peonidin-based anthocyanins on the probiotic bacteria proliferation

The numbers of Bifidobacterial strains (*B*. *infantis*, *B*. *adolescentis*, and *B*. *bifidum)* and *Lactobacillus acidophilus* (*L*. *acidophilus*) were represented as log cfu/mL in Table 2 after 48 h of *in vitro* incubation with P1, P2, P3, P4, P5, PSPAs and fructooligosaccharide (FOS). Compared with the proliferation promoting effect of FOS on probiotic bacteria, significant growth of the probiotic bacteria was observed at low concentrations of the various tested anthocyanins, however, the proliferation was clearly inhibited with the continuous increase of the anthocyanin concentrations. Together with the growth of *B*. *bifidum* as an example, the infection concentrations could be found at 1.5, 1.5, 1.0, 1.0, 1.0 and 1.0 mg/mL for PSPAs, P1, P2, P3, P4 and P5, respectively.

**Table 2:** Probiotic count in anaerobic fermentation medium containing different concentrations of PSPAs, P1, P2, P3, P4 and P5 at 37 °C for 48 h.

Samples	Concentration (mg/mL)	Probiotic count (log cfu/mL)			
		*B*. *bifidum*	*B*. *adolescentis*	*B*. *infantis*	*L*. *acidophilus*
FOS	0	0.38 ± 0.04^Ab^	0.32 ± 0.04^Ac^	0.42 ± 0.05^Ac^	0.48±0.07^Ab^
	0.5	2.10 ± 0.05^ABa^	1.94 ± 0.13^Bb^	1.88 ± 0.08^Bb^	2.33 ± 0.07^Aa^
	1.0	2.18 ± 0.05^ABa^	2.14 ± 0.09^Bab^	2.04 ± 0.06^Bab^	2.42 ± 0.10^Aa^
	1.5	2.31 ± 0.05^ABa^	2.24 ± 0.08^ABa^	2.15 ± 0.10^Ba^	2.44 ± 0.09^Aa^
	2.0	2.17 ± 0.12^ABa^	2.20 ± 0.07^ABab^	2.07±0.06^Bab^	2.42 ± 0.11^Aa^
	2.5	2.17 ± 0.07^Ba^	2.17 ± 0.07^Bab^	2.02 ± 0.06^Bab^	2.41 ± 0.07^Aa^
PSPAs	0	0.41 ± 0.04^Ac^	0.31 ± 0.06^Bb^	0.41 ± 0.05^Ab^	0.50 ± 0.04^Ab^
	0.5	0.94 ± 0.03^ABb^	0.85 ± 0.04^Bab^	0.93 ± 0.04^ABa^	1.06 ± 0.05^Aab^
	1.0	1.05 ± 0.03^Ba^	0.99 ± 0.05^BCa^	0.92 ± 0.06^Ca^	1.11 ± 0.06^Aa^
	1.5	1.11 ± 0.04^Aa^	0.94±0.03^Ba^	0.91 ± 0.04^Ba^	1.13 ± 0.05^Aa^
	2.0	1.02 ± 0.05^ABa^	0.86 ± 0.05^Bab^	0.91 ± 0.06^Ba^	1.10 ± 0.06^Aa^
	2.5	0.93 ± 0.09^Bb^	0.82 ± 0.05^Cab^	0.89 ± 0.07^BCa^	1.05 ± 0.05^Aab^
P1	0	0.40 ± 0.05^Ab^	0.30 ± 0.06^Bc^	0.42 ± 0.06^Ac^	0.49 ± 0.05^Ab^
	0.5	1.43 ± 0.05^Bab^	1.36 ± 0.04^Bb^	1.39 ± 0.06^Bb^	1.70 ± 0.06^Aab^
	1.0	1.61 ± 0.07^Bab^	1.51 ± 0.06^BCa^	1.46 ± 0.05^Ca^	1.75 ± 0.07^Aa^
	1.5	1.69 ± 0.06^Aa^	1.54 ± 0.05^Ba^	1.40 ± 0.04^Ba^	1.73 ± 0.07^Aa^
	2.0	1.50 ± 0.05^Bab^	1.53 ± 0.04^Ba^	1.38 ± 0.07^Bb^	1.71 ± 0.06^Aa^
	2.5	1.41 ± 0.04^BCab^	1.50 ± 0.07^Ba^	1.36 ± 0.05^Cb^	1.68 ± 0.04^Aab^
P2	0	0.42 ± 0.07^Ac^	0.32 ± 0.07^Bb^	0.43 ± 0.07^Ab^	0.50 ± 0.06^Ac^
	0.5	1.08 ± 0.06^Ab^	0.81 ± 0.05^Bab^	0.90 ± 0.05^Ba^	1.09 ± 0.04^Ab^
	1.0	1.22 ± 0.06^Aa^	0.93 ± 0.06^Ba^	0.96 ± 0.04^Ba^	1.14 ± 0.06^Aab^
	1.5	1.21 ± 0.08^Aa^	0.89 ± 0.07^Cab^	1.02 ± 0.06^Ba^	1.18 ± 0.04^Aa^
	2.0	1.13 ± 0.06^Aab^	0.87 ± 0.05^Bab^	0.98 ± 0.06^Ba^	1.15 ± 0.05^Aab^
	2.5	1.07 ± 0.04^Ab^	0.85 ± 0.07^Bab^	0.97 ± 0.06^Aa^	1.11 ± 0.06^Aab^
P3	0	0.44 ± 0.06^Ab^	0.31 ± 0.05^Ab^	0.42 ± 0.06^Ab^	0.48 ± 0.07^Ab^
	0.5	1.05 ± 0.03^Aab^	0.84 ± 0.06^Bab^	0.88 ± 0.05^Ba^	1.04 ± 0.04^Aab^
	1.0	1.18 ± 0.04^Aa^	0.88 ± 0.04^Ba^	0.90±0.05^Ba^	1.13 ± 0.05^Aa^
	1.5	1.16 ± 0.06^Aa^	0.89 ± 0.06^Ba^	0.92 ± 0.06^Ba^	1.11 ± 0.05^Aa^
	2.0	1.12 ± 0.04^Aa^	0.87 ± 0.08^Ba^	0.88 ± 0.05^Ba^	1.09 ± 0.06^Aab^
	2.5	1.07±0.05^Aab^	0.84 ± 0.06^Bab^	0.86 ± 0.07^Ba^	1.05 ± 0.05^Aab^
P4	0	0.41 ± 0.04^Bc^	0.32 ± 0.06^Bb^	0.43±0.04^Bb^	0.51 ± 0.06^Ab^
	0.5	0.72 ± 0.03^Bb^	0.64±0.06^Cab^	0.71 ± 0.05^Ba^	0.82 ± 0.04^Aab^
	1.0	0.87 ± 0.02^Aa^	0.70 ± 0.05^Ba^	0.73 ± 0.06^Ba^	0.90±0.05^Aa^
	1.5	0.86 ± 0.04^Aa^	0.74 ± 0.04^Aa^	0.72 ± 0.08^Aa^	0.85 ± 0.05^Aab^
	2.0	0.78 ± 0.03^Aab^	0.70 ± 0.06^Aa^	0.71 ± 0.05^Aa^	0.83 ± 0.05^Aab^
	2.5	0.74 ± 0.04^Aab^	0.67 ± 0.06^Bab^	0.69 ± 0.05^Ba^	0.79 ± 0.04^Aab^
P5	0	0.42 ± 0.05^Bb^	0.31 ± 0.05^Bb^	0.42±0.06^Bb^	0.51 ± 0.06^Ab^
	0.5	0.74 ± 0.03^Aab^	0.62 ± 0.03^Bab^	0.68 ± 0.04^Ba^	0.78 ± 0.04^Aab^
	1.0	0.85 ± 0.03^Aa^	0.71 ± 0.06^Ba^	0.70 ± 0.05^Ba^	0.82±0.05^Aa^
	1.5	0.79 ± 0.04^Aab^	0.69 ± 0.07^Ba^	0.68 ± 0.06^Ba^	0.80±0.05^Aa^
	2.0	0.75 ± 0.03^Aab^	0.67 ± 0.08^Ba^	0.66 ± 0.07^Ba^	0.76±0.07^Aab^
	2.5	0.73 ± 0.03^Aab^	0.65 ± 0.07^Bab^	0.65 ± 0.07^Ba^	0.73 ± 0.07^Aab^

Data were expressed as means ± SD. Different capital letters indicate significant differences (*P* < *0*.*05*) in the probiotic counts among different bacteria. Different lowercase letters indicate significant differences (*P* < *0*.*05*) in the probiotic count among different concentrations.

Meanwhile, as shown in Table 3, the significant decreases of the pH values in the media were obtained in the proliferation processes of *B*. *bifidum*, *B*. *adolescentis*, *B*. *infantis* and *Lactobacillus acidophilus* with the supplementation of peonidin-based anthocyanins at 0.5 mg/mL, and the changes were similar to the variations in the bacterial counts.

**Table 3:** The pH values of mediums in anaerobic fermentation for probiotics containing different concentrations of PSPA, P1, P2, P3, P4 and P5 at 37 °C for 48h.

Samples	Concentration (mg/mL)	pH values of mediums			
		*B*. *bifidum*	*B*. *adolescentis*	*B*. *infantis*	*L*. *acidophilus*
FOS	0	6.58 ± 0.19^Aa^	6.44 ± 0.15^ABa^	6.48 ± 0.14^ABa^	6.11 ± 0.21^Ba^
	0.5	4.40 ± 0.11^Ab^	4.18 ± 0.18^Bb^	4.45 ± 0.12^Aab^	4.29 ± 0.18^Bab^
	1.0	4.36 ± 0.09^Ab^	4.05 ± 0.18^Bc^	4.30 ± 0.15^Ab^	4.15 ± 0.18^Bb^
	1.5	4.27 ± 0.12^ABb^	4.12 ± 0.13^Bb^	4.38 ± 0.14^Aab^	4.30 ± 0.14^Aab^
	2.0	4.38 ± 0.07^Ab^	4.15 ± 0.13^Bb^	4.42 ± 0.18^Aab^	4.28 ± 0.19^ABab^
	2.5	4.37 ± 0.08^Ab^	4.16 ± 0.08^Bb^	4.36 ± 0.28^Ab^	4.31 ± 0.10^ABab^
PSPAs	0	6.53 ± 0.18^Aa^	6.41 ± 0.20^Aa^	6.48 ± 0.26^Aa^	6.17 ± 0.26^Ba^
	0.5	4.88 ± 0.19^Aab^	4.99 ± 0.18^Bb^	4.91 ± 0.16^Ab^	4.85 ± 0.13^Aab^
	1.0	4.76 ± 0.18^Bb^	4.83 ± 0.20^Abc^	4.83 ± 0.28^Ab^	4.71 ± 0.16^Bb^
	1.5	4.71 ± 0.22^Bb^	4.79 ± 0.14^Bc^	4.90 ± 0.22^Ab^	4.77 ± 0.24^Bb^
	2.0	4.86 ± 0.24^Aab^	4.86 ± 0.19^Abc^	4.85 ± 0.14^Ab^	4.78 ± 0.18^Bb^
	2.5	4.87 ± 0.21^ABab^	4.81 ± 0.20^Bbc^	4.93 ± 0.17^Ab^	4.82 ± 0.18^Bab^
P1	0	6.48 ± 0.14^Aa^	6.47 ± 0.30^Aa^	6.52 ± 0.23^Aa^	6.15 ± 0.30^Ba^
	0.5	4.73 ± 0.12^Aab^	4.40 ± 0.14^Bb^	4.66 ± 0.16^ABab^	4.64 ± 0.18^ABb^
	1.0	4.61 ± 0.11^Ab^	4.34 ± 0.22^Bb^	4.54 ± 0.27^Ab^	4.55 ± 0.24^Ab^
	1.5	4.68 ± 0.08^Ab^	4.20 ± 0.20^Bb^	4.57 ± 0.18^Ab^	4.52 ± 0.11^ABb^
	2.0	4.73 ± 0.14^Aab^	4.30 ± 0.17^Bb^	4.60 ± 0.24^Ab^	4.65 ± 0.30^Ab^
	2.5	4.69 ± 0.10^Ab^	4.35 ± 0.17^Bb^	4.58 ± 0.23^ABb^	4.57 ± 0.17^ABb^
P2	0	6.55 ± 0.19^Aa^	6.46 ± 0.20^ABa^	6.54 ± 0.12^Aa^	6.33 ± 0.18^Ba^
	0.5	4.90 ± 0.17^Aab^	4.73 ± 0.22^Bb^	4.97 ± 0.16^Ab^	4.82 ± 0.21^ABb^
	1.0	4.78 ± 0.20^Ab^	4.61 ± 0.16^Bb^	4.84 ± 0.20^Ab^	4.71 ± 0.28^ABb^
	1.5	4.72 ± 0.23^ABb^	4.61 ± 0.20^Bb^	4.85 ± 0.21^Ab^	4.75 ± 0.21^ABb^
	2.0	4.80 ± 0.11^Bb^	4.69 ± 0.23^Bb^	4.93 ± 0.24^Ab^	4.81 ± 0.24^Bb^
	2.5	4.88 ± 0.22^Aab^	4.60 ± 0.18^Bb^	4.88 ± 0.17^Ab^	4.76 ± 0.19^ABb^
P3	0	6.51 ± 0.14^Aa^	6.49 ± 0.16^Aa^	6.55 ± 0.20^Aa^	6.31 ± 0.21^Ba^
	0.5	4.94 ± 0.14^Aab^	4.74 ± 0.27^Bb^	4.98 ± 0.21^Aab^	4.93 ± 0.15^Aab^
	1.0	4.81 ± 0.27^Ab^	4.60 ± 0.17^Bb^	4.84 ± 0.16^Ab^	4.81 ± 0.16^Ab^
	1.5	4.87 ± 0.20^ABb^	4.63 ± 0.24^Bb^	4.93 ± 0.28^Aab^	4.88 ± 0.17^Ab^
	2.0	4.81 ± 0.26^Bb^	4.73 ± 0.11^Cb^	4.85 ± 0.28^Bb^	4.92 ± 0.23^Aab^
	2.5	4.92 ± 0.13^Aab^	4.64 ± 0.13^Bb^	4.96 ± 0.26^Aab^	4.86 ± 0.18^Ab^
P4	0	6.49 ± 0.13^Aa^	6.47 ± 0.27^Aa^	6.51 ± 0.15^Aa^	6.34 ± 0.20^Ba^
	0.5	5.06 ± 0.23^Bb^	4.98 ± 0.26^Bb^	5.21 ± 0.22^Aab^	5.05 ± 0.06^Bab^
	1.0	5.03 ± 0.14^ABb^	4.86 ± 0.09^Bb^	5.10 ± 0.27^Ab^	4.97 ± 0.20^ABb^
	1.5	4.96 ± 0.25^Bc^	4.89 ± 0.21^Bb^	5.16 ± 0.32^Ab^	4.92 ± 0.14^Bb^
	2.0	5.06 ± 0.25^ABb^	4.96 ± 0.19^Bb^	5.21 ± 0.32^Aab^	5.02 ± 0.19^Bab^
	2.5	5.12 ± 0.25^Aab^	4.85 ± 0.32^Bb^	5.13 ± 0.27^Ab^	4.98 ± 0.22^ABb^
P5	0	6.47 ± 0.09^Aa^	6.51 ± 0.17^Aa^	6.49 ± 0.12^Aa^	6.24 ± 0.23^Ba^
	0.5	4.94 ± 0.25^Bc^	4.92 ± 0.23^Bb^	5.18 ± 0.24^Ab^	5.02 ± 0.22^ABb^
	1.0	4.91 ± 0.14^ABc^	4.83 ± 0.16^Bb^	5.10 ± 0.28^Ab^	5.09 ± 0.15^Ab^
	1.5	5.04 ± 0.16^Bb^	4.89 ± 0.12^Cb^	5.02 ± 0.20^Bb^	5.17 ± 0.30^Aab^
	2.0	5.15 ± 0.20^Aab^	4.94 ± 0.23^Ab^	5.10 ± 0.15^Ab^	5.09 ± 0.17^Ab^
	2.5	5.05 ± 0.28^Ab^	4.89 ± 0.12^Bb^	5.08 ± 0.22^Ab^	5.04 ± 0.30^Ab^

Data were expressed as means ± SD. Different capital letters indicate significant differences (*P* < 0.05) in the probiotic count among different bacteria. Different lowercase letters indicate significant differences (*P* < 0.05) in the probiotic count among different concentrations.

### Effects of peonidin-based anthocyanins on the growth of harmful bacteria

**Figure 4:**
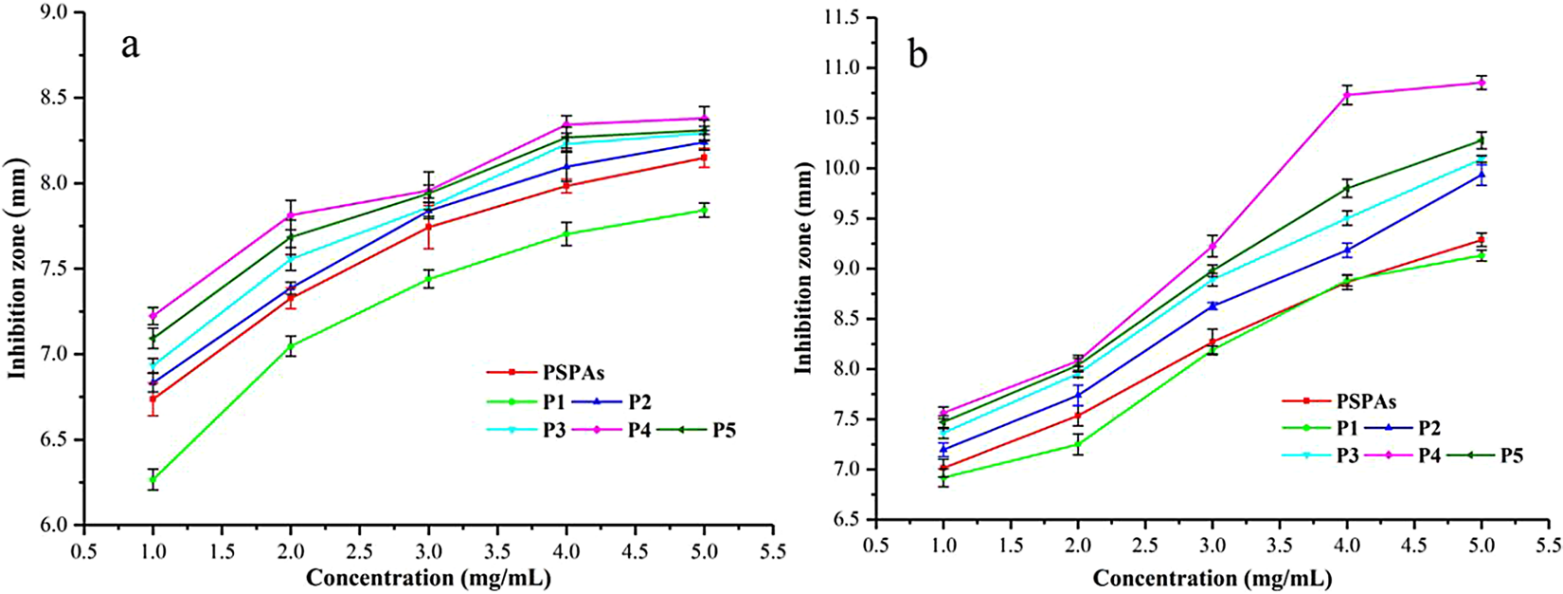
The inhibitory effect of PSPAs and peonidin-based anthocyanin monomers (P1, P2, P3, P4 and P5) on (**a**) *Staphylococcus aureus*, (**b**) *Salmonella typhimurium*, respectively. Data were obtained from three independent experiments and represented as mean values.

The antibacterial properties of five peonidin-based anthocyanins on *Staphylococcus aureus* and *Salmonella typhimurium* were evaluated by the filter paper method (Fig. 4). The sigmoidal curves for the diameter of the inhibition zone were observed over a concentration range of 1–5 mg/mL, and each anthocyanin fraction demonstrated a significant inhibitory effect on the growth of *Staphylococcus aureus* and *Salmonella typhimurium*, respectively, compared with the control without anthocyanin (0 mm). The sizes of the inhibition zones increased with the increase of the concentration of the samples, and the inhibition activity on the growth of *Staphylococcus aureus* and *Salmonella typhimurium* was in the order of P4 > P5 > P3 > P2 > PSPAs > P1. In addition, compared to *Staphylococcus aureus*, the antibacterial activity of peonidin-based anthocyanins on *Salmonella typhimurium* was more effective.

The minimum inhibitory concentration (MIC) was established in Table 4 as the lowest concentration of anthocyanin that inhibited the visible growth of the pathogenic bacteria from the intestinal tract. *Staphylococcus aureus* and *Salmonella typhimurium* were inhibited by the anthocyanins with MIC values ranging from 0.25 to 0.50 mg/mL, and high inhibitory effects of the anthocyanins against Gram-positive and Gram-negative strains are presented in the P4 and P5 groups at 0.25 mg/mL, followed by P3 (0.50 and 0.25 mg/L, respectively).

**Table 4:** Minimal inhibitory concentrations (MIC) of PSPAs, P1, P2, P3, P4 and P5 on *Staphylococcus aureus* and *Salmonella typhimurium* at 37 °C for 12 h.

strains	MIC (mg/mL)					
	PSPAs	P1	P2	P3	P4	P5
*Staphylococcus aureus*	0.50 ± 0.06	0.75 ± 0.09	0.50 ± 0.08	0.50 ± 0.09	0.25 ± 0.03	0.25 ± 0.07
*Salmonella typhimurium*	0.50 ± 0.04	0.50 ± 0.04	0.50 ± 0.06	0.25 ± 0.06	0.25 ± 0.05	0.25 ± 0.04

Data were obtained from three independent experiments and expressed as means ± SD.

## Discussion

Various anthocyanins contents have been found in different PSP cultivars. The total anthocyanin content from *Ipomoea batatas L*. was calculated to be 13.73 ± 0.13 mg/100 g PSP, which was similar to the anthocyanin content of 12.40 mg/100 g of the Japanese ‘Purple Sweet’ variety peonidin-type^15^, and it confirmed the effectiveness of the extraction method. Since few qualitative and quantitative studies on the anthocyanins from PSP cultivars in China have been reported, the anthocyanin profile analysis based on UV and MS is important.

The abundance differences of each peak between HPLC (Fig. 1(a)) and preparative HPLC (Fig. 1(b)) resulted from the variability in the chromatographic conditions. As shown in Fig. 1(a) and Table 1, ten anthocyanins were acylated with *p*-hydroxybenzoic acid, caffeic acid and/or ferulic acid, except the anthocyanins of peaks 1 and 2. Meanwhile, two kinds of glycosides, sophoroside and glucoside, were found to be connected with all twelve anthocyanins. And the twelve anthocyanin peaks were tentatively identified as two major kinds of compounds, cyanidin-based anthocyanins and peonidin-based anthocyanins, due to the characteristic MS-MS fragments at m/z 287 and 301, respectively^13^. Interestingly, all the main peaks (2, 4, 6, 10, 11 and 12) were peonidin-types (Figs 2 and S1–S4), and the total content of the peonidin-based anthocyanins was approximately four-fold higher than the cyanidin-based anthocyanins, which was similar to the peonidin/cyanidin ratio of 4.52 calculated from the new Japanese cultivar, ‘Purple Sweet’, and the new American variety, ‘Stokes Purple’^16^.

A number of researchers have shown a higher correlation between individual anthocyanin and biological activities because of the type and content^5^. To our knowledge, a series of cyanidin-based anthocyanin *in vitro* antioxidant activities have been investigated according to the structural variations^17^. However, few studies have been published regarding to antioxidant and prebiotic properties of the most abundant peonidin-based anthocyanins that exist in PSP. Furthermore, the higher acylated ratio in the current study was also a potential suggestion for high activity^5^.

As expected, the five peonidin-based anthocyanins were ranked as P4 (peak 11) > P5 (peak 12) > P3 (peak 10) > P2 (peak 6) > P1 (peak 2) in the free radical-scavenging activity analysis at a low concentration (60 μg/mL) (Fig. 3(a)), which corresponded with the structure characteristics. The scavenging activity of anthocyanins could be explained by the donation of hydrogen, and it has been well known that di-acylated anthocyanins possess higher antioxidant acidity than the mono-acylated and non-acylated anthocyanins^2^.

Recently, much attention has been focused on the potential scavenging activity of anthocyanins against reactive oxygen and nitrogen species, which might prevent cell damage. Similar to the results of the DPPH scavenging activity analysis, the superoxide anion radical scavenging activity of five anthocyanin compounds was in the order of P4 > P5 > P3 > P2 > P1 (Fig. 3(b)), which was attributed to the chemical structure-activity relationship. The presence of a hydroxyl group at C-3 and the higher phenolic acids content, such as *p*-hydroxybenzoic acid, caffeic acid and ferulic acid, enhanced the O^2−^ scavenging effect^18^.

As indicated in Fig. 3(c), although the reducing power of Vc was significantly higher, all the tested anthocyanin samples showed more higher potent activities at low concentrations in the microgram range. And the variations in the ability to quench radicals by electron donation might be due to the total phenolic acids content^19^.

Because of the catalysis of transition metals in lipid peroxidation, hydroxyl radicals (OH·) would be generated due to oxidative damage. Therefore, the chelating activity of ferrous ion is important to form chelates with the transition metal ion^20^. The rank of the metal chelating activities of the tested anthocyanins (Fig. 3(d)) was similar to the antioxidant activities in the superoxide anion radical scavenging activity and total reducing power activity. Although few reports on the metal chelating activity have previously been published, our results suggest that the spatial conformation, position and number of electron-donating ligating groups in anthocyanin compounds could result in a higher metal chelating activity^21^.

A concentration-dependent analysis was carried out to evaluate the effect of five peonidin-based anthocyanins and PSPAs on the proliferation of probiotic bacteria. Interestingly, our results (Table 2) did not follow the stronger dose-response correlation for the cyanidin-based anthocyanins as previously reported^22^. According to the published results, the catabolism of peonidin-based anthocyanins would be favorable for bacterial growth, while cytoplasm pyknosis and disintegration could also be induced by high phenolic acid content through the reaction with the mycoproteins and enzymes of the strains^23^.

As shown in Table 3, the decrease in the pH at an anthocyanin concentration of 0.5 mg/mL was accompanied by the proliferation of probiotic bacteria, which indicated that anthocyanins would be deglycosylated by β-glucosidases that were secreted through probiotics, and the resulting glucose could be utilized by the bacteria. Meanwhile, phenolic/organic acid could be generated by the degradation of the de-glycosylated anthocyanins which could adjust the environmental pH for bacterial growth^24^. Our results also showed that the higher concentrations of peonidin-based anthocyanins might inhibit the nutrition intake of the tested probiotic bacteria, and anthocyanins could be utilized by varying degrees depending on the bacterial species.

Furthermore, the acylated anthocyanins exhibited a higher inhibition sensitivity on harmful bacteria (Fig. 4, Table 4). According to the previous reports, the propagation and spread as well as the nutrition intake of harmful bacteria could be limited by anthocyanins, and the cellular metabolism of pathogens could be disturbed by anthocyanin metabolites^25^.

## Methods

### Material and chemicals

Purple sweet potato (PSP, *Ipomoea batatas* (L.) *Lam*.) was obtained from a local supermarket, which was cultured in the suburb of Hefei city, Anhui Province, China. Two separation membranes (molecular weight cut-off 3 and 0.2 kDa, respectively) were provided by Huakang Membrane Co., Ltd. (Hefei, China). Macroporous resin X-5 was obtained from Samsung Resin Technology Co., Ltd. (Jiangsu, China). Procyanidin was purchased from Bomei Bio-technology Co., Ltd. (Hefei, China). HPLC-grade acetonitrile and methanol were purchased from Tedia Co., Ltd. (Shanghai, China). Additionally, 1,1-diphenyl-2-picrylhydrazyl (DPPH), trifluoroacetic acid, ethanol, citric acid, hydrogen peroxide and other analytical grade chemicals were obtained from Sinopharm Chemical Reagent Co., Ltd. (Shanghai, China). MRS basal medium (pH 7.0), BA medium (pH 6.8), BS medium (pH 7.2), TSA medium (pH 7.3) and TSB medium (pH 7.3) were purchased from Beijing Luqiao Co., Ltd. (Beijing, China).

*Bifidobacterium bifidum* (CICC 6071), *Bifidobacterium adolescentis* (CICC 6070), *Bifidobacterium infantis* (CICC 6069), *Lactobacillus acidophilus* (CICC 6096), *Staphylococcus aureus* (CICC 10201) and *Salmonella* (CICC 10420) were obtained from Chinese Collection Center of Industrial Culture (CICC). The stock cultures were stored in broth containing 10% glycerol at −80 °C.

### Extraction of anthocyanins

Previous procedures with minor modifications were applied to extract anthocyanins from PSP^26^. One hundred grams of PSP were cleaned and cut into small strips (3 mm × 1 mm), and then ground in a colloid mill (JM, Shanghai Aisijie Co., Ltd., China) with 1.5 L extracting solvent, which contained 3.5% citric acid and 79 U/mL cellulose. The samples were subsequently incubated at 55 °C for 2 h, and followed by centrifugation at 4000 rpm for 20 min. The extraction process was repeated three times, and all the extracts were filtered and concentrated by separation membranes with 3 and 0.2 kDa molecular weight cut-offs, respectively. Then, a macroporous resin X-5 column was applied for the further purification at a flow rate of 2.0 mL/min. The column was washed with three column volumes (3 CV) of deionized water and eluted with 4 CV of ethanol (80:20, V/V). The eluent was dialyzed and freeze-dried till the water content was less than 5%. Then, the PSPAs were obtained and stored at −20 °C for further experiments.

### Determination of total anthocyanin content

A modified pH differential method was utilized to determine the total anthocyanin content^4^. Briefly, 1.0 mg/mL PSPAs was prepared and aliquoted in duplicate. One duplicate was diluted with hydrochloric acid-potassium chloride buffer (0.2 M, pH 1.0), and the other was diluted with sodium acetate buffer (0.2 M, pH 4.5). After equilibration for 15 min, the absorbance of each sample was measured at wavelengths of 522 nm and 700 nm, respectively, by a UV-Vis spectrophotometer (Precision Scientific Instrument Co., Ltd., Shanghai, China) with pure water as a blank. The absorbance was calculated as follows:

1 $${\rm{A}}={({A}_{522}-{A}_{700})}_{pH1.0}-{({A}_{522}-{A}_{700})}_{pH4.5}$$

The total anthocyanin content was calculated and expressed as cyanidin-3-glucoside equivalents using the following equation:

2 $${\rm{C}}={\rm{A}}\times {\rm{MW}}\times \frac{{\rm{DF}}}{{\rm{\varepsilon }}\times {\rm{L}}}\times {\rm{V}}$$

Where C was the total anthocyanin content calculated as cyanidin-3-glucoside (mg/100 g PSP), V is the collection volume prior to freeze drying, MW was the molecular weight of cyanidin-3-glucoside (449.2 g/mol), DF was the dilution factor, ε was the molar absorbance of cyanidin-3-glucoside (26900 L/cm/mol), and L was the cell path length (1 cm).

### HPLC analysis and LC-MS/MS identification

PSPAs were dissolved in pure water containing 0.05% hydrochloric acid at a concentration of 1 mg/mL, and passed through a 0.20 µm filter membrane (Jinteng Experiment Equipment Co., Ltd., Tianjin, China) prior to HPLC analysis using a Waters apparatus (2410, Waters Co., Ltd., USA) equipped with a 2598 UV-Vis detector and connected to a 4.6 mm × 250 mm C18 column (Waters Co., Ltd., USA). The temperature of the column was set as 30 °C, and the flow rate and injection volume were 0.8 mL/min and 20 µL, respectively. The anthocyanins were detected at 520 nm and eluent A was 0.05% trifluoroacetic acid in water (V/V), while eluent B was acetonitrile. The elution process was 10% B for 0–5 min, 10–12% B for 5–10 min, 12–16% B for 10–15 min, 16–17% B for 15–20 min, 17–19% B for 20–25 min, 19–20% B for 25–30 min, 20–30% B for 30–35 min, 30–40% B for 35–40 min, 40–90% B for 40–42 min, 90–10% B for 48–50 min, and 10% B for 50–55 min,.

The LC-MS/MS was subsequently used to identify the monomeric components of PSPAs. Mass spectrometry was performed with an Impact II Q-TOF MS/MS (Bruker Daltonics, Billerica, MA, USA). Mass spectra in the m/z range of 200–1500 were obtained by electrospray ionization in positive-ion mode. The experimental conditions were as follows: ESI interface, 45 psi; dry gas (nitrogen), 50.0 psi, 0.5 L/min, dry temperature 340 °C.

### Purification of five peonidin-based anthocyanin monomers from PSP

Five peonidin-based anthocyanin monomers from PSP were purified using preparative high-performance liquid chromatography (Preparative HPLC) (Lisui Technology Co., Ltd., Suzhou, China) connected with a Galaksil EP-C18 column (20 mm × 250 mm, 10 μm). Five milliliters of PSPAs were injected at a concentration of 20 mg/mL. The temperature of the column was set at 30 °C and the flow rate was 12 mL/min. The anthocyanins were detected at 520 nm using eluent A (0.05% trifluoroacetic acid) and B (acetonitrile) as the mobile phase. The solvent gradient was 10% B for 8 min, 10–16% B for 8 min, 16–17% B for 8 min, 17–19% B for 8 min, 19–20% B for 10 min, 20–30% B for 15 min, and 30–40% B for 10 min. The elution peaks were collected by an automatic collector, and then freeze-dried till the water content was less than 5%. The purity of the five peonidins was characterized by the HPLC method.

### DPPH radical (DPPH·) scavenging capacity analysis

The DPPH· scavenging capacity was determined using the method described by Brand-Williams with some modifications^27^. Two milliliters of test samples were mixed with 2 mL of 20 mM DPPH· solution in ethanol. Then, the mixtures were shaken and placed at room temperature for 30 min before measuring the absorbance at 517 nm (A_1_). Distilled water was used to replace the DPPH· solution and test samples, which were blank one and two, respectively. The DPPH· scavenging capacity was calculated as follows:

3 $${\rm{Scavening}}\,{\rm{ability}}( \% )=[1-({{A}}_{1}-{{A}}_{2})/{{A}}_{0}]\times 100 \% $$

Where A_2_ and A_0_ were the absorbance of blank one and two, respectively.

### Superoxide anion (O^2−^·) scavenging capacity analysis

The pyrogallol autoxidation method was applied to determine the O^2−^· scavenging capacity. Two milliliters of the test samples and 75 μL of 45 mM pyrogallol were successively mixed with 2.25 mL of 50 mM Tris-HCl buffer solution (pH 8.2). Then, 20 μL of 10 M HCl were added for termination after a 4-min reaction, and the absorbance of the mixture was measured at 320 nm (A_1_). Distilled water was used to replace the pyrogallol and test samples as blank one and two, respectively. The O^2−^· scavenging capacity was calculated as follows:

4 $$\text{Scavening}\,\text{ability}\,( \% )=[1-({{A}}_{1}-{{A}}_{0})/{{A}}_{2}]\times 100 \% $$

Where A_0_ and A_2_ were the absorbance of blank one and two, respectively.

### Total reducing power (TRP) analysis

A ferricyanide/Prussian blue method was performed to analyze the total reducing power activity. The phosphate buffer solution (2.5 mL, 0.2 M, pH 6.6) and 2.5 mL of 1% potassium ferricyanide solution were successively mixed with 1 mL of the test samples. Then, the mixtures were incubated at 50 °C for 20 min. Upon cooling to room temperature, 2.5 mL of the mixture was taken out and mixed with 2.5 mL of 10% trichloroacetic acid, 0.5 mL of 0.1% ferric trichloride solution and 2.5 mL water. Reducing the power activity (A) was represented by the absorbance of the mixture measured at 700 nm after a 10-min incubation at room temperature.

### Ferrous ion-chelating activity analysis

Ferrous ion-chelating activity was evaluated according to the method of Dinis with some modification^28^. A sample (2 mL) was mixed with 0.05 mL 2 mM FeCl_2_ solution, and 0.2 mL 5 mM Ferric ion solution was added. After a 10-min incubation at room temperature, the absorbance of the mixture was measured at 562 nm (A_1_), and the distilled water sample was set as the blank without metal chelating activity. The ferrous ion-chelating activity (F) was calculated as follows:

5 $${\rm{F}}( \% )=[({A}_{0}-{A}_{1})/{A}_{0}]\times 100 \% $$

Where A_0_ was the absorbance of the blank.

### Effects of five peonidin-based anthocyanins on the probiotic bacteria proliferation

The Bifidobacterial strains (*B*. *infantis*, *B*. *adolescentis*, and *B*. *bifidum)* and *Lactobacillus acidophilus* were used to investigate the prebiotic activity of the peonidin-based anthocyanins. The Bifidobacterial strains and *Lactobacillus acidophilus* were activated with BS and MRS medium, respectively, according to the method established by Huebner^29^. The samples were added to the culture mediums at concentrations of 0, 0.5, 1.0, 1.5, 2.0, or 2.5 mg/mL, respectively, with fructooligosaccharide (FOS) as the control. After recording the initial total bacterial count, the media was incubated in an anaerobic incubator (YQX-III, Shanghai Wanrui Laboratory Equipment Co., Ltd. Shanghai, China) at 37 °C for 36 h with a culture condition of 85% N_2_, 10% H_2_ and 5% CO_2_. Then, the count of the probiotics and the pH of each sample were measured. For each sample, serial dilutions of the fluid medium were prepared with sterile 1% (W/V) peptone solution and duplicated 100 μL aliquots were spread on MRS agar plates. After anaerobic incubation at 37 °C for 48 h, the bacteria count was rescored as log cfu/mL.

### Effects of five peonidin-based anthocyanins on the growth of harmful bacteria

The antibacterial activity of the peonidin-based anthocyanins on the growth of *Staphylococcus aureus* and *Salmonella typhimurium* were detected by the filter paper method. The pathogenic bacteria were activated with TSB medium in a constant temperature shaker (HASUC, Shanghai union instrument manufacturing Co., Ltd. Shanghai, China) at 37 °C and 180 r/min till the OD value was 0.5, and 100 μL of the bacteria suspension was added to the petri dishes and coated evenly under aseptic conditions. The filter papers disks (Whatman No. 1, 6 mm diameter) containing 15 μL of each sample at different concentrations (1, 2, 3, 4 and 5 mg/mL, respectively) were put in the center of the petri dishes. The diameter of the resulting zone of inhibition was measured after a 12 h incubation at 37 °C.

The minimum inhibitory concentration (MIC) of the peonidin based anthocyanins against the growth of *Staphylococcus aureus* and *Salmonella typhimurium* was determined. Two-fold serial dilutions of anthocyanins were mixed with culture media to get a final concentration ranging from 25 to 3000 μg/mL. The bacterial suspension was inoculated onto each plate and incubated at 37 °C for 12 h, including on a growth control without anthocyanins and a sterility control without bacterial suspension. The MIC was defined as the lowest concentration of anthocyanins that prevented the visible growth of the bacteria^30^.

### Statistical analysis

Each experiment was carried out in triplicate. One-way analysis of variance and Tukey’s comparison of the means were performed using IBM SPSS Statistical software (version 20, Chicago, IL, USA), and a difference of *P* < 0.05 was considered significant.

## Conclusions

The main anthocyanins from the Chinese cultivar PSP (*Ipomoea batatas* (L.) *Lam*.) were identified as the peonidin types. Five peonidin-based anthocyanin monomers exerted stronger *in vitro* antioxidant activities. And the peonidin-based anthocyanin monomers might be potential natural probiotic sources that could increase the proliferation of *Bifidobacterial strains (B*. *infantis*, *B*. *adolescentis*, and *B*. *bifidum)* and *Lactobacillus acidophilus*, as well as inhibit the growth of the intestinal pathogens of *Staphylococcus aureus* and *Salmonella typhimurium*. The results revealed the potential benefits for the consumption of the Chinese cultivar PSP in the human diet, and the characteristics of anthocyanin monomers will be useful for functional foods and pharmaceutical developments.

## Electronic supplementary material

Supplementary information
