# Supplementary material for: Antioxidant and prebiotic activity of five peonidin-based anthocyanins extracted from purple sweet potato (Ipomoea batatas (L.) Lam.)
Source: Sci Rep. 2018 Mar 22;8:5018. doi: 10.1038/s41598-018-23397-0 (PMC5864876; doi:10.1038/s41598-018-23397-0)
Supplement: Supplementary file 1 — Supplementary information [file 41598_2018_23397_MOESM1_ESM.docx]

Supplementary information belonging to

**Antioxidant and** **prebiotic activity of five** **peonidin-based anthocyanins extracted from** **purple sweet potato (*Ipomoea batatas* (L.) *Lam.*)**

Hanju Sun ^a,1^, Pingping Zhang ^a,1^, Yongsheng Zhu ^a^, Qiuyan Lou ^a^, Shudong He ^a,b^*,

^a^ School of Food Science and Engineering, Hefei University of Technology, Hefei 230009, Anhui, PR China

^b^ School of Food Science and Technology, National Engineering Research Center of Seafood, Dalian Polytechnic University, Dalian 116034, PR China

^1^ Hanju Sun and Pingping Zhang contributed equally to this work.

* Corresponding author. Tel: + (86) 551-62901505; fax: + (86) 551-62901516 (S. He)

E-mail address: * shudong.he@hfut.edu.cn (S. He)


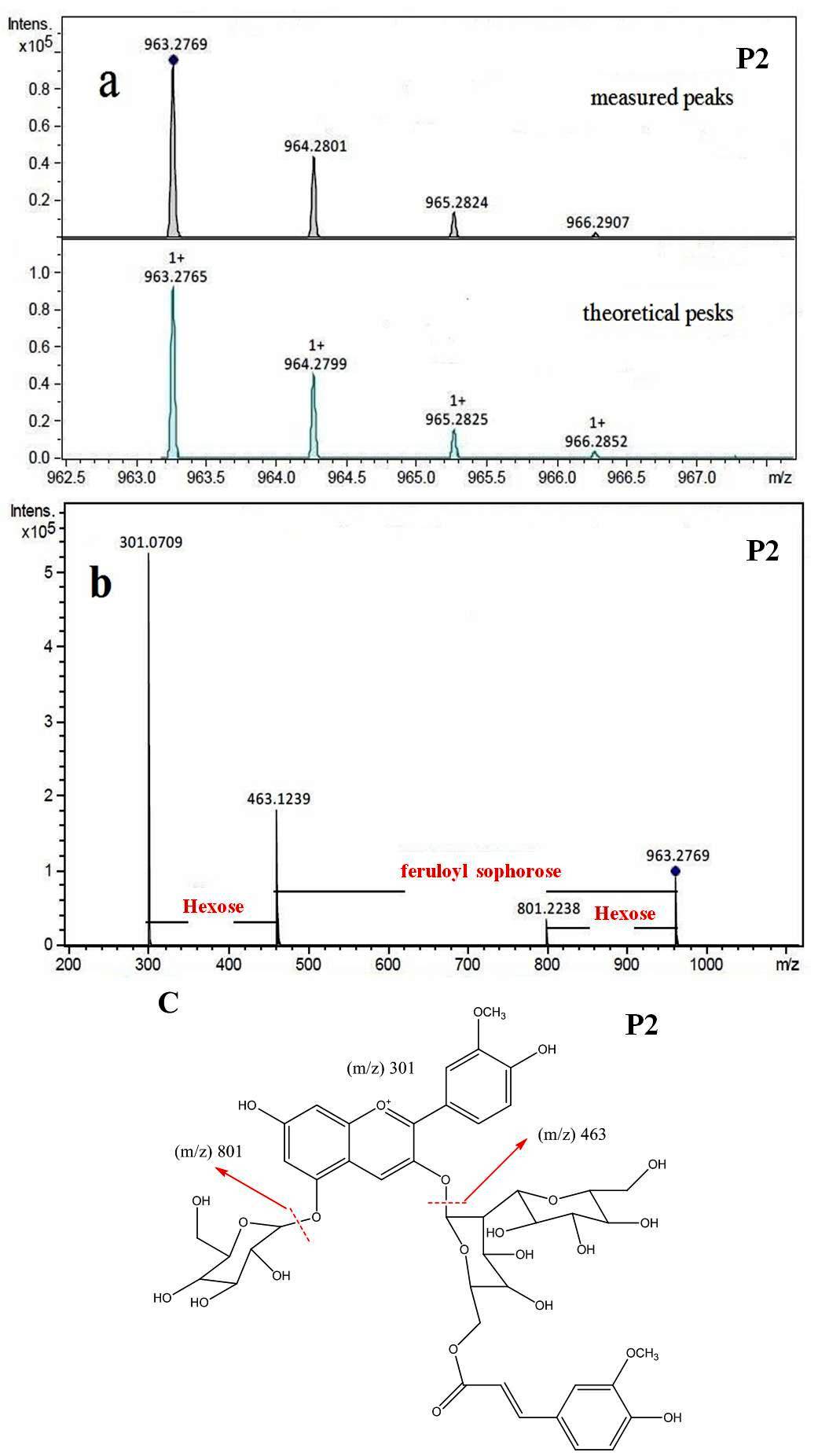


**Supplementary Figure S1.** Mass spectrometric data and chemical structure of peonidin-based anthocyanins. **(a)** HPLC-TOF-MS spectrum of P2 (peak 6), **(b)** HPLC-TOF-MS/MS spectrum of P2 (peak 6), **(c)** chemical structure of P2 (peak 6).


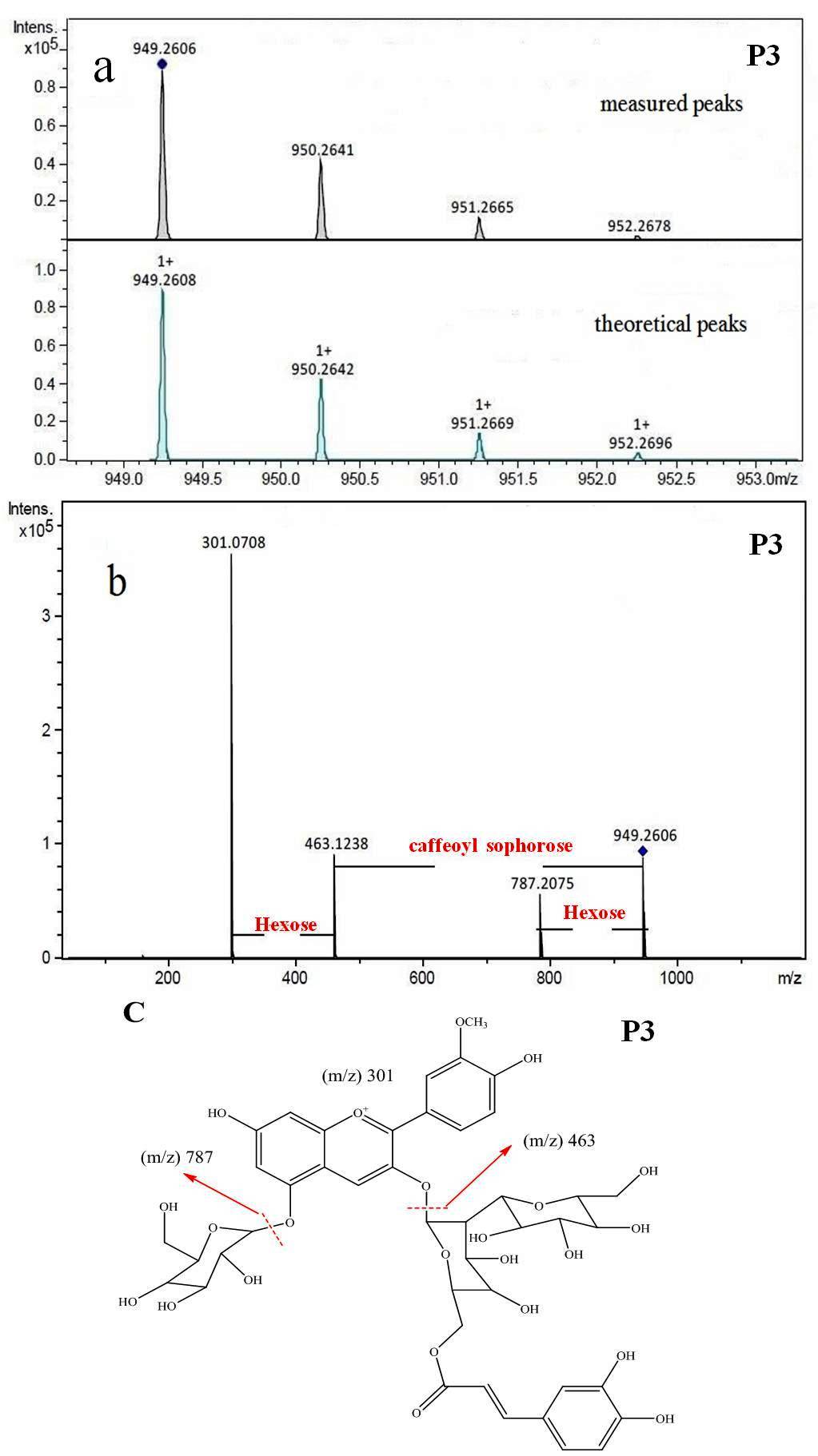


**Supplementary Figure S2.** Mass spectrometric data and chemical structure of peonidin-based anthocyanins. (a) HPLC-TOF-MS spectrum of P3 (peak 10), (b) HPLC-TOF-MS/MS spectrum of P3 (peak 10), (c) chemical structure of P3 (peak 10).


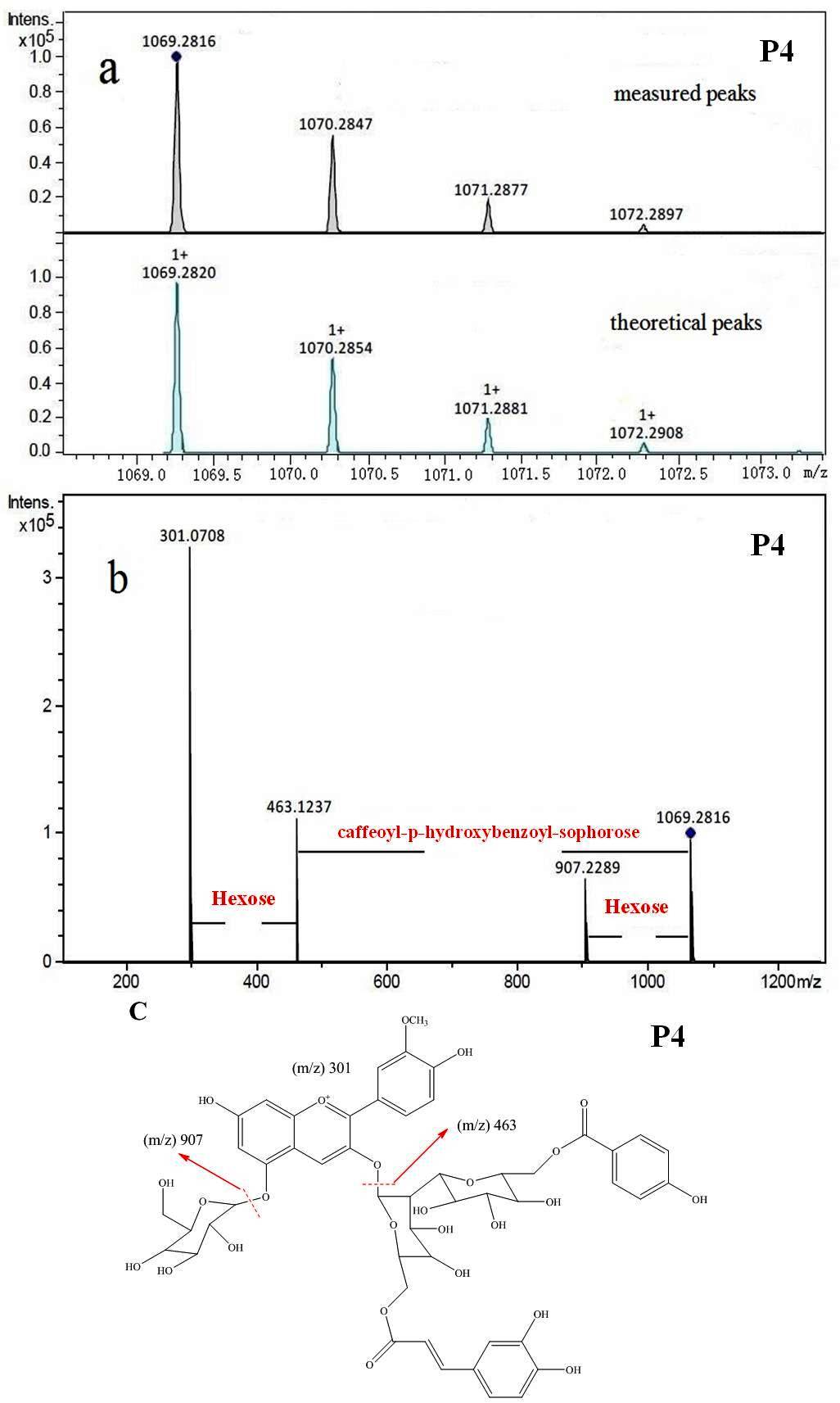


**Supplementary Figure S3.** Mass spectrometric data and chemical structure of peonidin-based anthocyanins. (a) HPLC-TOF-MS spectrum of P4 (peak 11), (b) HPLC-TOF-MS/MS spectrum of P4 (peak 11), (c) chemical structure of P4 (peak 11).


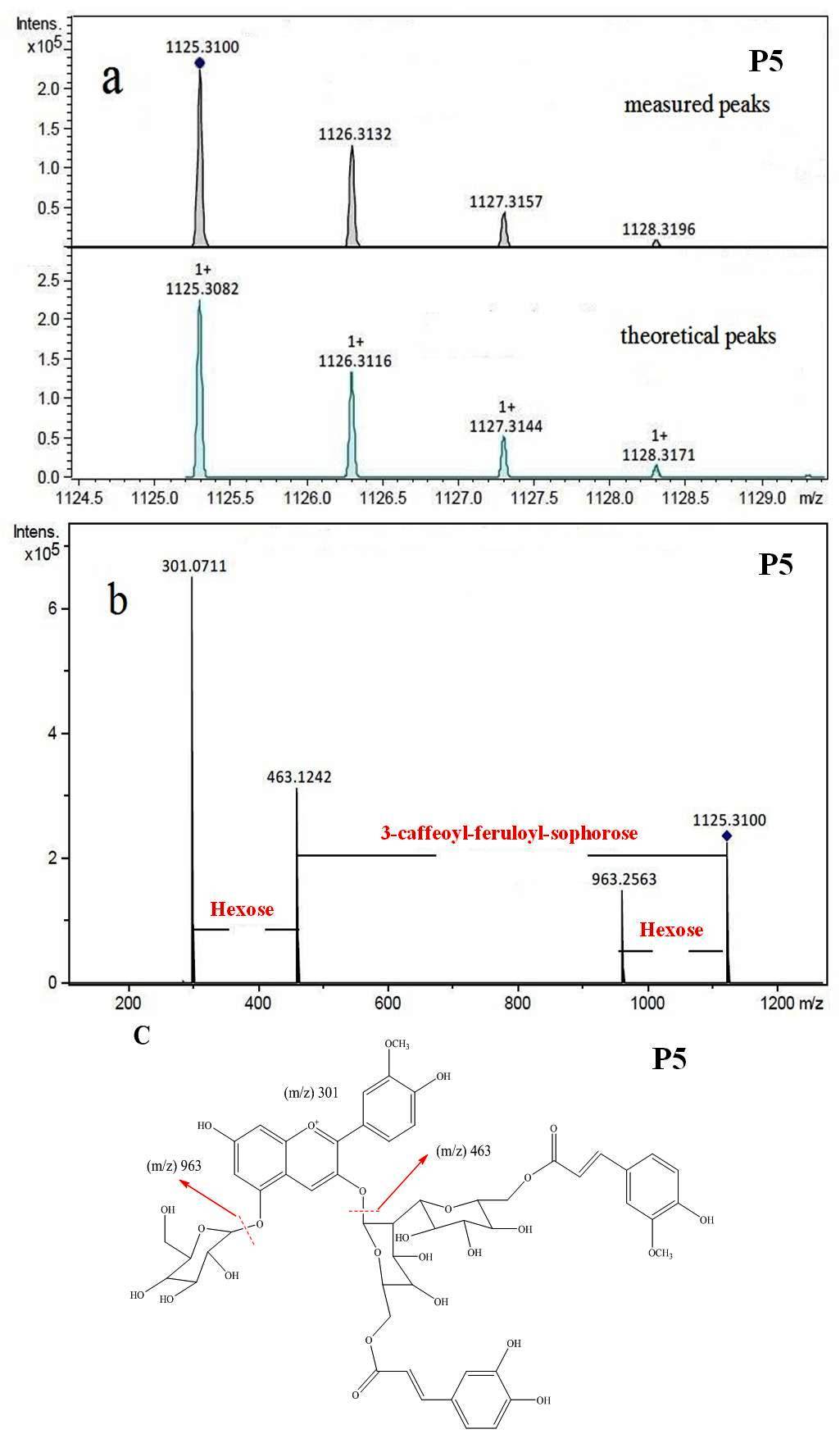


**Supplementary Figure S4.** Mass spectrometric data and chemical structure of peonidin-based anthocyanins. (a) HPLC-TOF-MS spectrum of P5 (peak 12), (b) HPLC-TOF-MS/MS spectrum of P5 (peak 12), (c) chemical structure of P5 (peak 12).
